# Genetic Diversity of the Invasive Gall Wasp *Leptocybe invasa* (Hymenoptera: Eulophidae) and of its *Rickettsia* Endosymbiont, and Associated Sex-Ratio Differences

**DOI:** 10.1371/journal.pone.0124660

**Published:** 2015-05-13

**Authors:** Francesco Nugnes, Marco Gebiola, Maurilia Maria Monti, Liberata Gualtieri, Massimo Giorgini, Jianguo Wang, Umberto Bernardo

**Affiliations:** 1 Istituto per la Protezione Sostenibile delle Piante, Consiglio Nazionale delle Ricerche, Portici (NA), Italy; 2 Department of Entomology, The University of Arizona, Tucson, Arizona, the United States of America; 3 Department of Plant Protection, College of Agriculture, Jiangxi Agricultural University, Nanchang, Jiangxi Province, China; University of Minnesota, UNITED STATES

## Abstract

The blue-gum chalcid *Leptocybe invasa* Fisher & LaSalle (Hymenoptera: Eulophidae) is a gall wasp pest of *Eucalyptus* species, likely native to Australia. Over the past 15 years it has invaded 39 countries on all continents where eucalypts are grown. The worldwide invasion of the blue gum chalcid was attributed to a single thelytokous morphospecies formally described in 2004. Subsequently, however, males have been recorded in several countries and the sex ratio of field populations has been found to be highly variable in different areas. In order to find an explanation for such sex ratio differences, populations of *L*. *invasa* from a broad geographical area were screened for the symbionts currently known as reproductive manipulators, and both wasps and symbionts were genetically characterized using multiple genes. Molecular analyses suggested that *L*. *invasa* is in fact a complex of two cryptic species involved in the rapid and efficient spread of the wasp, the first recovered from the Mediterranean region and South America, the latter from China. All screened specimens were infected by endosymbiotic bacteria belonging to the genus *Rickettsia*. Two closely related *Rickettsia* strains were found, each infecting one of the two putative cryptic species of L. *invasa* and associated with different average sex ratios. *Rickettsia* were found to be localized in the female reproductive tissues and transovarially transmitted, suggesting a possible role of *Rickettsia* as the causal agent of thelytokous parthenogenesis in *L*. *invasa*. Implications for the variation of sex ratio and for the management of *L*. *invasa* are discussed.

## Introduction

*Leptocybe invasa* Fisher & La Salle (Hymenoptera: Eulophidae: Tetrastichinae) commonly known as blue-gum chalcid is a gall wasp of many *Eucalyptus* species. It was unknown until the early 2000s, when it was recorded in Italy, identified as *Aprostocetus* sp. [1], and in Turkey [2]. Successively it was described as *L*. *invasa* and reported as an invasive pest in the Palearctic region [3]. Due to its exclusive association with *Eucalyptus*, the blue-gum chalcid is believed to be native to Australia [3], where some parasitoids monophagous on *L*. *invasa* have also been recorded [4]. However, its origin remains unproven [5] because there are also a small number of *Eucalyptus* species native to New Guinea, Indonesia and Philippines [6,7] and there are some records of phytophagous insects that have shifted onto eucalypts and achieved pest status out of Australia [8]. Nevertheless, there is consensus that there has been a major radiation of eulophid gall inducers in Australia [9], and several species of Australian gall-inducing eulophids have become invasive within the last decade [10].

Although destructive and rapid insect invasions are widely reported [11–15], the scale and the speed of *L*. *invasa* invasion have few precedents [16,17]. Blue-gum chalcid seems to show wide ecological plasticity and indeed, in about 15 years, has spread worldwide (39 countries, spanning Europe, North Africa, Middle East, Asia, South America, Oceania and North America, S1 Table), and its invasiveness, based on the terminology in [18], reached stage V, becoming a dominant pest species [5].

*Leptocybe invasa* lays eggs in plant tissues causing the formation of galls on the leaf midribs and petioles and on the stem of new shoots, eventually leading to leaf-curling and premature aging of the leaves [3]. Egg overloading might cause death of juvenile shoots, while severe attacks lead to leaf fall, stunted growth and may eventually seriously weaken the tree [3]. In recently invaded countries, *L*. *invasa* can compromise the productivity of *Eucalyptus* plantations [17,19]. Conversely, *L*. *invasa* is not considered detrimental for *Eucalyptus* plantations in Australia, because the natural enemies are able to control its populations to tolerable levels [4]. Different species of *eucalyptus* show different susceptibility to *L*. *invasa* attacks [3] with *E*. *grandis* W. Hill, *E*. *camaldulensis* Dehnh and *E*. *tereticornis* Smith being the most susceptible [20].

The eucalyptus gall wasp was originally described as a thelytokous species [3], as field populations were represented only by females. Afterwards, males were recorded and described in some populations from Turkey [21], India [22], China [23], Thailand [24] and Taiwan [25] with a sex ratio ranging from 0.5% males in Turkish populations [21] up to 18–48% in Chinese populations [26]. Female-biased sex ratio occurs frequently in Hymenoptera [27] and, in chalcidoid lineages especially, it has been associated with infection of symbiotic intracellular bacteria able to manipulate their host’s reproduction by inducing male-killing (mortality of infected male embryos), feminization (genotypic diploid males develop as functional phenotypic females from unfertilized eggs) or more commonly thelytokous parthenogenesis (mothers produce only diploid daughters from unfertilized haploid eggs) [28–32]. In the family Eulophidae, in particular, parthenogenesis induced by bacterial endosymbionts has been documented in three different species, the *Wolbachia*-infected *Tetrastichus coeruleus* Nees [32], and the *Rickettsia*-infected *Neochrysocharis formosa* (Westwood) [29] and *Pnigalio soemius* (Walker) [33].

Here we studied populations of *L*. *invasa* from countries where only females have been reported (Italy, Argentina, Tunisia) ([1,34,35] S2 Table) and from countries where males are also present (Turkey, China) [21,26]. This study aims at ascertaining if: 1) cryptic species are involved in the invasive process of *L*. *invasa*; 2) the sex ratio of *L*. *invasa* is related to infection by bacterial endosymbionts. Therefore, genetic variability of *L*. *invasa* was assessed by sequencing nuclear and mitochondrial genes of different geographic populations. These populations were also screened for infection by endosymbionts, and symbiont diversity was in turn characterized by sequencing four different bacterial genes. Fluorescent in Situ Hybridization (FISH) was used to localize endosymbionts within the hosts and to test for vertical transmission.

## Materials and Methods

### Ethics Statement

The sampling of living material involved in our experiments included wasps, i.e. *Leptocybe invasa*, associated with galls on *Eucalyptus* sp. All sampling locations are not privately owned or protected (coordinates in Table 1). Besides neither the host plant nor the wasp species are endangered or otherwise protected, and therefore no specific permits were required for these locations/activities.

**Table 1:** Specimens investigated and analyses performed in the study.

Code	Locality	Longitude	Latitude	Altitude m a.s.l.	Host	Sex	PCR Rbf Rbr	Symbiont detected in DGGE	Genbank Accession Code							
									Insect				*Rickettsia*			
									28S-D2	COI 5’ region	COI 3’ region	ITS2	16S	*gltA*	*atpA*	*rpmE* tRNAf^Met^
*Li*_IT_1	Portici, Italy	14°21’05” N	40°48’55” E	29	*E*. *camaldulensis*	♀	+	*Rickettsia*	KP143969	KP233972		KP143943	KP233904	KP233935	KP233920	KP233955
*Li*_IT_2						♀	+	*Rickettsia*	KP143970	KP233973		KP143944	KP233905	KP233936	KP233921	KP233956
*Li*_IT_3						♀	+	*Rickettsia*	KP143971	KP233974		KP143945	KP233906	KP233937	KP233922	-
*Li*_IT_4						♀	+	*Rickettsia*	KP143972	KP233975		KP143946	-	KP233938	-	-
*Li*_IT_5						♀	+	*Rickettsia*	KP143973	KP233976		KP143947	-	KP233939	-	-
*Li*_IT_6	S. M. al Bagno, Italy	17°59’49” N	40°07’33” E	4	*E*. *camaldulensis*	♀	+	*Rickettsia*	-	KP233977		-	-	-	-	-
*Li*_IT_7						♀	+	*Rickettsia*	-	KP233978		-	-	-	-	-
*Li*_IT_10	Costa Saracena Sicily, Italy	15°07’31” N	37°18’32” E	22	*E*. *camaldulensis*	♀	+	*Rickettsia*	-	KP233989	-	KP143948	KP233907	KP233940	KP233923	-
*Li*_AR_1	La Plata, Argentina	57°56’20” S	34°54’17” W	31	*E*. *camaldulensis*	♀	+	*Rickettsia*	KP143974	KP233979		KP143949	KP233908	KP233941	KP233924	KP233957
*Li*_AR_2*						♀	+	*Rickettsia*	KP143975	KP233980		KP143950	KP233909	KP233942	KP233925	KP233958
*Li*_AR_3						♀	+	*Rickettsia*	KP143976	KP233981		KP143951	KP233910	KP233943	KP233926	-
*Li*_CN_1*	Hubian, Gangzhou City, Jiangxi Province, China	114°54’38” N	25°53’16” E	142	*E*. *globulus*	♀	+	*Rickettsia*	KP143987	KP233985		KP143962	KP233911	KP233944	KP233927	KP233964
*Li*_CN_2						♂	+	*Rickettsia*	KP143988	KP233986		KP143963	KP233912	KP233945	KP233928	KP233965
*Li*_CN_3						♀	+	*Rickettsia*	KP143989	KP233987		KP143964	KP233913	KP233946	-	-
*Li*_CN_4						♀	+	*Rickettsia*	KP143990	KP233988		KP143965	KP233914	KP233947	KP233929	-
*Li*_CN_5						♂	+	*Rickettsia*	KP143991	-	KP233994	KP143966	-	-	-	-
*Li*_CN_6						♀	+	-	KP143992	KP233990	-	KP143967	-	-	-	-
*Li*_CN_7*						♀	+	-	KP143993	KP233991	-	KP143968	-	-	-	-
*Li*_CN_8*						♀	+	-	KP143994	KP233992	-	-	-	-	-	-
*Li*_CN_9*						♀	+	-	KP143995	KP233993	-	-	-	-	-	-
*Li*_TK_1*	Serinyol, Antakya, Hatay Province, Turkey	36°12’48” N	36°22’00” E	120	*E*. *camaldulensis*	♀	+	*Rickettsia*	KP143977	KP233953	KP233966	KP143955	KP233918	KP233951	KP233933	KP233961
*Li*_TK_2						♀	+	*Rickettsia*	KP143978	KP233954	KP233967	KP143956	KP233919	KP233952	KP233934	KP233962
*Li*_TK_3						♀	+	*Rickettsia*	KP143979	-	-	KP143957	-	-	-	-
*Li*_TK_4						♂	+	*Rickettsia*	KP143980	-	-	KP143958	-	-	-	KP233963
*Li*_TK_5						♂	+	*Rickettsia*	KP143981	-	-	KP143959	-	-	-	-
*Li*_TK_6						♀	+	*Rickettsia*	KP143982	-	-	KP143960	-	-	-	-
*Li*_TK_7						♀	+	*Rickettsia*	KP143983	-	-	KP143961	-	-	-	-
*Li*_TU_1	Mouaden, Tunisia	09°16’00” N	37°09’53” E	80	*E*. *camaldulensis*	♀	+	*Rickettsia*	KP143984	KP233982		KP143952	KP233915	KP233948	KP233930	KP233959
*Li*_TU_2						♀	+	*Rickettsia*	KP143985	KP233983		KP143953	KP233916	KP233949	KP233931	KP233960
*Li*_TU_3						♀	+	*Rickettsia*	KP143986	KP233984		KP143954	KP233917	KP233950	KP233932	-
*Bs*1	Portici, Italy	14°21’05” N	40°48’55” E	29	Ex *Bactrocera oleae* on *Olea europea europea*	♀	-	-	KP233970	KP233995		-	-	-	-	-
*Am*1	Bari, Italy	16°52’00” N	41°07’31” E	5	*Melilotus indicus*	♀	-	-	KP233969	KP233971		KP233968	-	-	-	-

*Li*: *Leptocybe invasa*; *Bs*: *Baryscapus silvestrii*; *Am*: *Aprostocetus monacoi*; +: specimen *Rickettsia* sp. positive in specific PCR;-: not determined.

*: destructive DNA extraction.

### Insect sampling

Stands of *Eucalyptus camaldulensis* Dehnhardt or *E*. *globulus* Labillardière infested with *L*. *invasa* were sampled from Portici, S. Maria al Bagno and Siracusa (Italy), La Plata (Argentina) and Hubian (China). Additional material was received from Antakya (Turkey) and Mouaden (Tunisia) (Table 1). Leaves harbouring galls of different ages were removed from the trees, placed in plastic boxes and stored at room temperature until the emergence of adults. Soon after their emergence, wasps were killed in 95% ethanol and morphologically identified using the original species description of females [3] and males [21].

### Molecular characterization and phylogenetic analyses of *L*. *invasa*

DNA was extracted from whole single individuals (males and females) by using both destructive and non-destructive Chelex and proteinase K based methods modified as in [36]. In this latter case, wasps were then treated and card mounted as in [37]. Three genes were sequenced: the mitochondrial cytochrome c oxydase subunit I (COI), and two ribosomal genes, the expansion segment D2 of the 28S ribosomal subunit (28S-D2) and the Internal Transcribed Spacer 2 (ITS2). COI is a very effective and universally used marker for species delimitation [38]. ITS2 has been successfully used at the species level in many Chalcidoidea taxa, e.g. Pteromalidae [39], Trichogrammatidae [40] and Eulophidae [37]. 28S-D2 has also proved diagnostic at the species level in Pteromalidae [39], Aphelinidae [41] and Eulophidae [36]. Two regions of COI were amplified, the first using the forward primer LCO1490 paired with either HCO2198 [42] or Lep-r1 [38] reverse primers, and the second pairing the forward primer C1-J-2183 [43] or Apf [44] with the reverse primer TL2-N-3014 [43]. The ribosomal gene 28S-D2 was amplified with primers D2F and D2R [39]. PCR reactions and cycling conditions for COI and 28S-D2 were set as described in [36]. For the amplification of ITS2, primers ITS2F [39] and ITS2TrichRev [40] were used in PCR reactions as in [45]. PCR products were checked on a 1.2% agarose gel stained with ethidium bromide and directly sequenced. Chromatograms were assembled, edited by eye and aligned with Bioedit 7.2.5 [46] and the sequences deposited in GenBank, with the accession numbers reported in Table 1.

All sequences with chromatograms showing ambiguous peaks were cloned. Amplicons were ethanol precipitated, ligated into the pGEM-T Easy plasmid vector (Promega), and cloned into *Escherichia coli* TOP10 competent cells (Invitrogen) according to the manufacturer’s instructions. Transformants were PCR-screened with universal M13 vector primers, and inserts of the expected size were sequenced. COI sequences were virtually translated into the corresponding amino acid chain to detect frame-shift mutations and stop codons, which may indicate the presence of pseudogenes [47,48].

Phylogenies for *L*. *invasa* were reconstructed using Bayesian inference (BI), utilizing MrBayes 3.1.2 [49], following the methodology reported in [37] on concatenated (ITS2–28S-D2–COI) alignment by using the GTR+G+I nucleotide model as selected by jModeltest [50]. Sharing some morphological features with *L*. *invasa*, two species of closely related genera, *Baryscapus silvestrii* Viggiani et Bernardo (Hymenoptera: Eulophidae) and *Aprostocetus monacoi* Viggiani (Hymenoptera: Eulophidae) were used as outgroups to root the tree. Sequence distances within and between populations of COI dataset were calculated as uncorrected pairwise distances (*p*-distances) using MEGA 4 [51]. We also looked for diagnostic single nucleotide polymorphisms (SNPs), i.e. character state in a given nucleotide position shared by all individuals from one group and different from all individuals in any other group, in COI sequences, as well as the occurrence of diagnostic non-synonymous amino acid changes.

For species delimitation, we used the Poisson tree processes model (PTP) [52], a recently developed method that been successfully applied [53]. All sequences of Tetrastichinae (5’-COI “barcoding” region) available in GenBank were included, aligned in BioEdit and run in MrBayes (GTR+G+I substitution model) for 1 million generations (rate sample = 1000, burn in value = 25%). BI tree obtained was run on the bPTP web server as in [53], for 500,000 MCMC generations. The relationships between *L*. *invasa* specimens were also investigated using Statistical Parsimony in TCS 1.21 [54] on the COI dataset, as in [55].

### Detection of bacterial symbionts

DGGE (Denaturing Gradient Gel Electrophoresis) analysis was used to check for the presence of bacterial symbionts in female and male specimens listed in Table 1. Bacterial 16S rRNA gene fragments were amplified by a nested PCR with the primers 341f (to which a 40-bp GC clamp was added) and 518r [56] using as template a longer fragment amplified with the primers 27F and 1513R [57] in a “touch-down” annealing protocol [58]. The following strains of arthropod reproductive manipulators were used as positive controls: *Wolbachia* from *Encarsia formosa* Gahan, *Cardinium* from *Encarsia pergandiella* Howard, *Rickettsia* from *P*. *soemius*, *Spiroplasma* from *Drosophila neotestacea* Grimaldi, James & Jaenike and *Arsenophonus* from *Bemisia tabaci* (Gennadius). PCR products were subjected to DGGE analysis slightly modifying the technique described in [59] (denaturing gradient 35–60% and 45–60%; 90V for 17h). In addition, to confirm DGGE results, diagnostic PCR was performed with specific primers targeting the 16S rRNA gene for *Cardinium* (CLOf and CLOr1 –[28]), *Rickettsia* (Rb-F and Rb-R—[60]), *Spiroplasma* (27F and TKSSsp—[61]) and *Arsenophonus* (27F and Ars16SR—[62]), and the *ftsZ* gene for *Wolbachia* (ftsZf1 and ftsZr1 –[63]).

### Molecular characterization of *Rickettsia*

Multiple genes were sequenced to characterize *Rickettsia* at the strain level. Approximately 1000 bp of the 16S rRNA gene were amplified with Rb-F and Rb-R primers as previously described; ~600 bp of the citrate synthase (*gltA*) gene were obtained by a nested PCR strategy with primers CS1d and CS1273r [64] followed by CS78d paired with CS715r [64]; the subunit α of ATP synthase F1 (*atpA*) gene (~750 bp) was amplified with ATP syn f1 α fw and ATP syn f1 α rev primers [65]. For both genes, amplifications were carried out using a “step-up” program: 5 min of initial denaturation at 94°C, first 10 cycles step at 94°C for 40 sec, 37°C for 2 min and 72°C for 90 sec and the second 35 cycles step with the same denaturation and elongation parameters and annealing temperature set at 48°C for 1 min. The amplification was completed by holding for 5 min at 72°C. Finally, the intergenic spacer *rpmE*-tRNAf^Met^, known to be useful for characterizing strains of *Rickettsia* [66], was sequenced using *rpmE*F and *rpmE*R primers [67]. All sequences were assembled, edited and aligned with Bioedit 7.2.5 [46], and deposited in GenBank, with the accession numbers reported in Table 1.

### *Rickettsia* phylogeny and host-endosymbiont relationship

Sequences of the 16S rRNA, *gltA* and *atpA* genes were aligned with homologous sequences available in GenBank. The related species *Orientia tsutsugamushi* (Hayashi) was included as outgroup in the 16S rRNA and *atpA* genes analyses [68], whereas a *Wolbachia* strain was used as outgroup in the *gltA* gene analysis. For *Rickettsia* 16s rRNA, *gltA* and *atpA* genes, phylogenetic reconstructions using Bayesian inference was performed using MrBayes 3.1.2 [49], following the methodology reported in [37]. Intergenic spacer region *rpmE*-tRNAf^Met^ sequences of *L*. *invasa* were compared to each other and checked against GenBank for highest similar matches.

To test for congruence between the host and endosymbiont trees, trees of *L*. *invasa* populations and of their own *Rickettsia* symbionts were compared. For this analysis, ML trees were built including *P*. *soemius* and its own *Rickettsia* sequences as outgroups, as the only eulophid taxon currently available in GenBank for which all five insect and symbiont genes here used are available.

### Fluorescence microscopy

Localization of *Rickettsia* within the host’s reproductive tissues was carried out, for the Italian population, using fluorescence in situ hybridization (FISH) with the *Rickettsia*-specific probe RickPn-Cy3 [33] and the universal probe EUB338-Cy5 [69]. Ovaries and mature eggs were extracted from adult females in a drop of PBS buffer under a stereomicroscope. Ovaries and eggs were subjected to the whole-mount FISH method reported by [33] except for the duration of egg dechorionation (10 min in 50% commercial bleach in PBS solution). Stained samples were observed both under a ZEISS Axiophot 2 epifluorescence microscope and under the Leica confocal TCS SP5 microscope. Images obtained on multiple focal planes were stacked with software Leica application Suite, Advances fluorescence 2.4.1. The specificity of the observed signals was verified with the following control experiments: no-probe control and RNase-digested control. Nuclei of the host cells were counterstained with DAPI (0.4 μg/ml) in mounting medium.

## Results

### Morphological characterization of *Leptocybe invasa*

All female and male specimens from different countries were identified as *L*. *invasa*, and did not show any appreciable difference in their morphology. Voucher material (listed in Table 1) is deposited at the Institute for Sustainable Plant Protection (IPSP, Portici, Italy).

### Molecular characterization and phylogenetic analyses of *L*. *invasa*

The ribosomal gene 28S-D2 sequences were obtained for 27 specimens (Table 1). Sequences ranged from 488 to 578 nt, and, after trimming, the final alignment consisted of 482 bp. The ribosomal gene 28S-D2 sequences of Italian, Tunisian, Argentinean and Turkish samples were identical to each other and differed by a single nucleotide from Chinese sequences (Table 2). Similarly, ITS2 sequences from Italian, Tunisian, Argentinean and Turkish samples were identical, whereas the Chinese sequences had a single missing nucleotide (427 bp versus 428 bp) (Table 2).

**Table 2:** Synthetic representation of the *L*. *invasa* and of its *Rickettsia* symbiont characterization.

	*Leptocybe invasa*				*Rickettsia* symbiont			
Populations	28S	ITS2	COI	Sex ratio %	16S	*gltA*	*rpmE*	*atpA*
Italy	a	a	a	0^1^ ^,^ ^2^ ^,^ ^3^	a	a	a	a
Argentina	a	a	a		a	a	a	a
Tunisia	a	a	a		a	a	a	a
Turkey	a	a	b	0.5^4^	a	a	a	a
	a	a	c		b	a	a	a
China	b	b	d	18–48^5^	c	b	b	a

Same letter indicates identical sequence respectively for each gene.

^1^This work.

^2^[35].

^3^[34].

^4^[21].

^5^[26].

Two regions of COI (1455 nucleotides in total) were sequenced from each of 19 specimens (Table 1). The same COI haplotype was recovered from the Italian, Argentinean and Tunisian populations, whereas one and two unique haplotypes were recovered from the Chinese and Turkish populations, respectively (Table 2). Clones of COI amplicons, that showed ambiguous peaks in the chromatograms of sequences from the Turkish population, mostly resulted from pseudogenes, except for two clones that were then used in subsequent analyses. BI phylogenetic analyses of the concatenated ITS2, 28S-D2 and COI (S1 Fig) showed two well-supported clades. The first clade includes *L*. *invasa* from China, and the second clade includes Italian, Argentinean and Tunisian populations (hereafter called lineage A) and the Turkish population.

**Fig 1:**
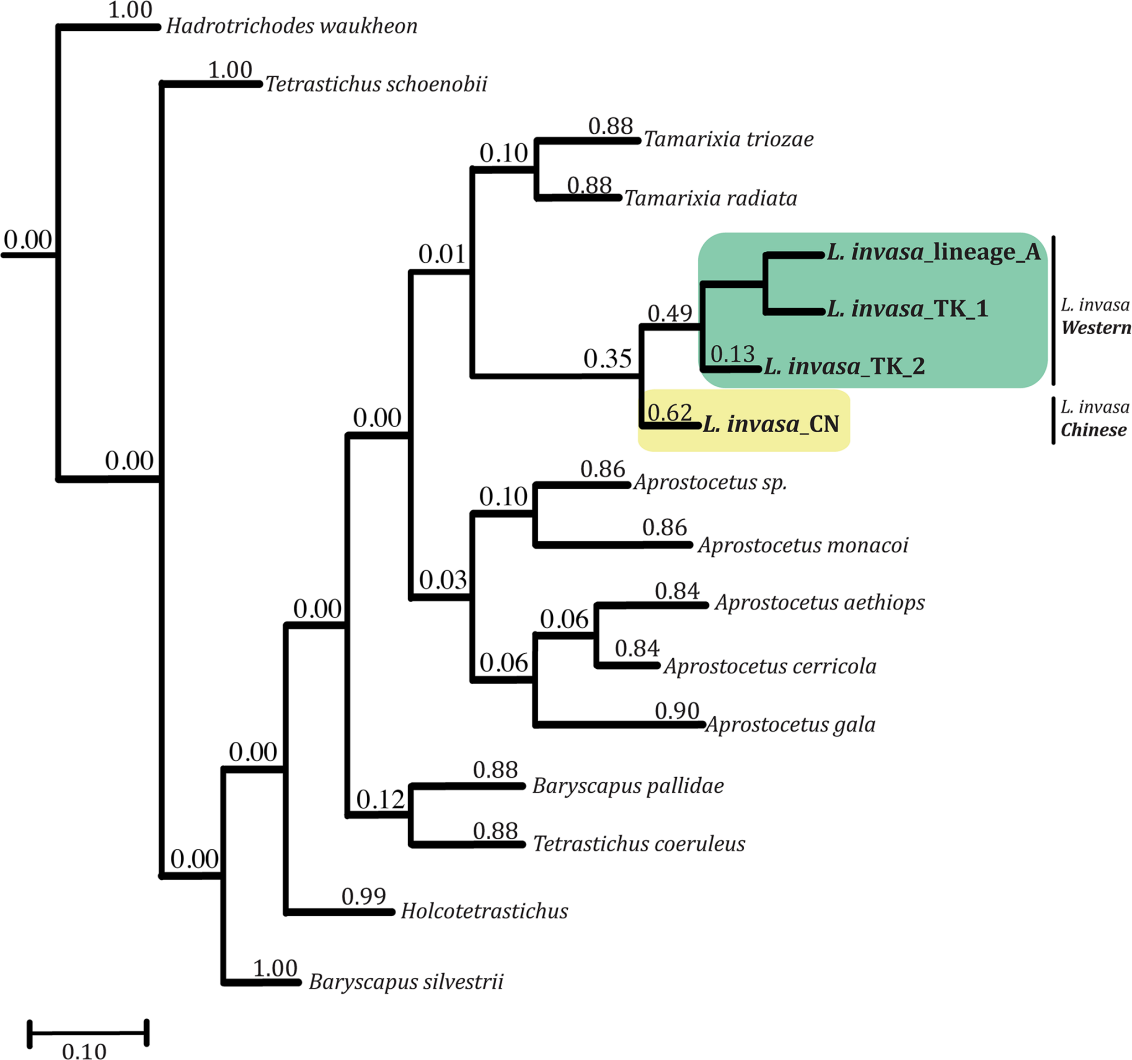
Species delimitation of *Leptocybe invasa* specimens based on bPTP analysis on the COI dataset. Clades highlighted with coloured boxes and names in bold after a | symbol correspond to recognised putative species of *L*. *invasa*. Posterior delimitation probabilities values are reported above branches.

The bPTP analysis indicates a moderate support for the existence of two cryptic species in the blue gum chalcid complex, delimiting *L*. *invasa* from China as a putative species (hereafter called *Chinese* lineage) and the monophyletic clade including lineage A and Turkish specimens as a second putative species (hereafter called *Western* lineage) (Fig 1). Based on this analysis, there is 65% probability that the four COI haplotypes do not represent a single species, 49% probability that the 3 haplotypes “lineage A”, “TK1” and “TK2” represent a single species (which is consistent with the slightly high uncorrected intra-specific distances in the Turkish population, see below) and 62% probability that the Chinese population is a separate species. Statistical parsimony on the COI dataset yielded two separate networks, corresponding to the *Western* and *Chinese* lineages. The connection limit necessary to obtain a single network was 48 steps.

The distance between Chinese and Turkish samples was 3.7%, that between Chinese lineage and lineage A was 3.1% (Table A in S1 File), and that between Chinese and Western lineages was 3.6% (Table B in S1 File). The two Turkish haplotypes resulted in an intraspecific distance of 1.5% (Table C in S1 File). Analysis of diagnostic sites for COI sequences showed that the Chinese lineage is distinguishable by 43 SNPs (S3 Table). Three diagnostic non-synonymous amino acid changes were found in the Chinese lineage: valine instead of isoleucine, methionine instead of isoleucine and serine instead of threonine.

### Detection of bacterial symbionts

DGGE analysis of PCR-amplified 16S rRNA gene showed that the only endosymbiont infecting all tested *L*. *invasa* adults (26 females and 4 males) was *Rickettsia* (data not shown). Congruent results were obtained by PCR screening, which revealed that 100% of individuals were infected only by *Rickettsia*.

### Molecular characterization of *Rickettsia*

*Rickettsia* 16S rRNA gene sequences (ranged from 744 to 837 bp) were obtained from 16 specimens (Table 1). No intra-lineage variation was found except within the Turkish population, where two sequences were recovered: one in specimen *Li*-TK1, shared with lineage A, and another found exclusively in specimen *Li*-TK2 (different by two nt). 16S rRNA gene of the *Chinese* lineage differed from all the others by two nt (Table 2). Sequences of the *Rickettsia gltA* gene (499 bp), obtained from 18 wasps (Table 1), were invariant, except for in the *Chinese* lineage, which differed by a single nucleotide. Sequences of the *atpA* gene (681 bp), obtained from 15 wasps (Table 1), were identical in *Rickettsia* found in all *L*. *invasa* specimens. *rpmE*-tRNAf^Met^ sequences (381 bp) were obtained from 11 wasps (Table 1). *Rickettsia* in individuals of the *Western* lineage showed the same sequence, which differed from the sequence found in *Rickettsia* in the individuals of the *Chinese* lineage by three nucleotides. The Chinese and all other samples sequences had a match of 97 and 98% identity with *R*. *felis rpmE*-tRNAf^Met^ sequences, respectively.

### *Rickettsia* phylogeny and host-endosymbiont relationship

**Fig 2:**
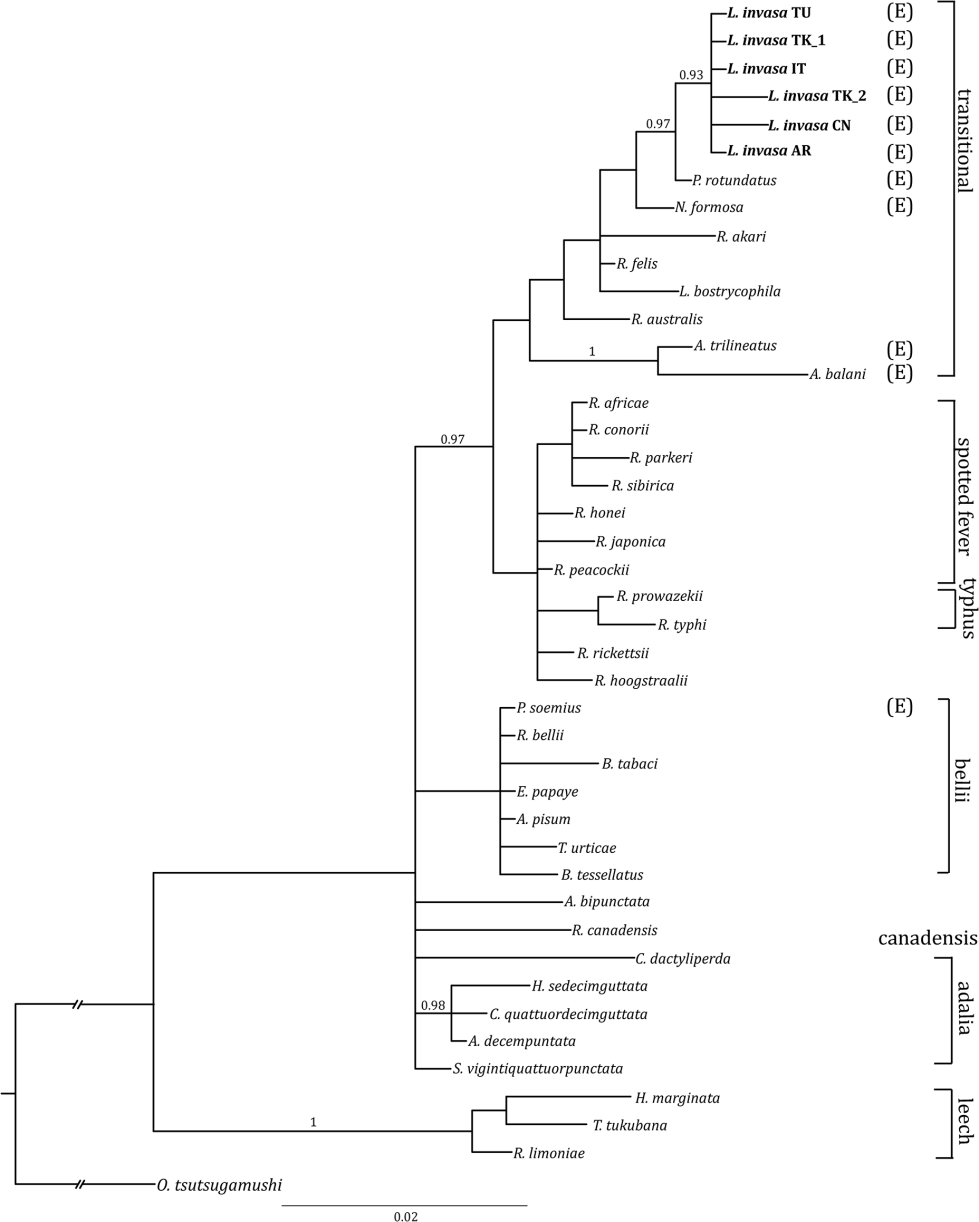
Bayesian majority-rule consensus tree based on the 16S *r*RNA dataset. BI tree shows the phylogenetic placement of the symbiont from *L*. *invasa* within the genus *Rickettsia*. The evolutionary model selected by MrModeltest2 was HKY+G. The host is provided whenever the symbiont is not identified at the species level. Posterior probabilities are reported above branches. Scale bar indicates the number of substitutions per site.

BI phylogenetic analyses of 16S rRNA, *gltA* and *atpA* genes showed that *Rickettsia* symbionts of *L*. *invasa* form a lineage in the *Rickettsia* transitional group (Fig 2 and S2 Fig). The host and symbiont trees have an identical topology, suggesting coevolution (S3 Fig).

### Fluorescence microscopy

**Fig 3:**
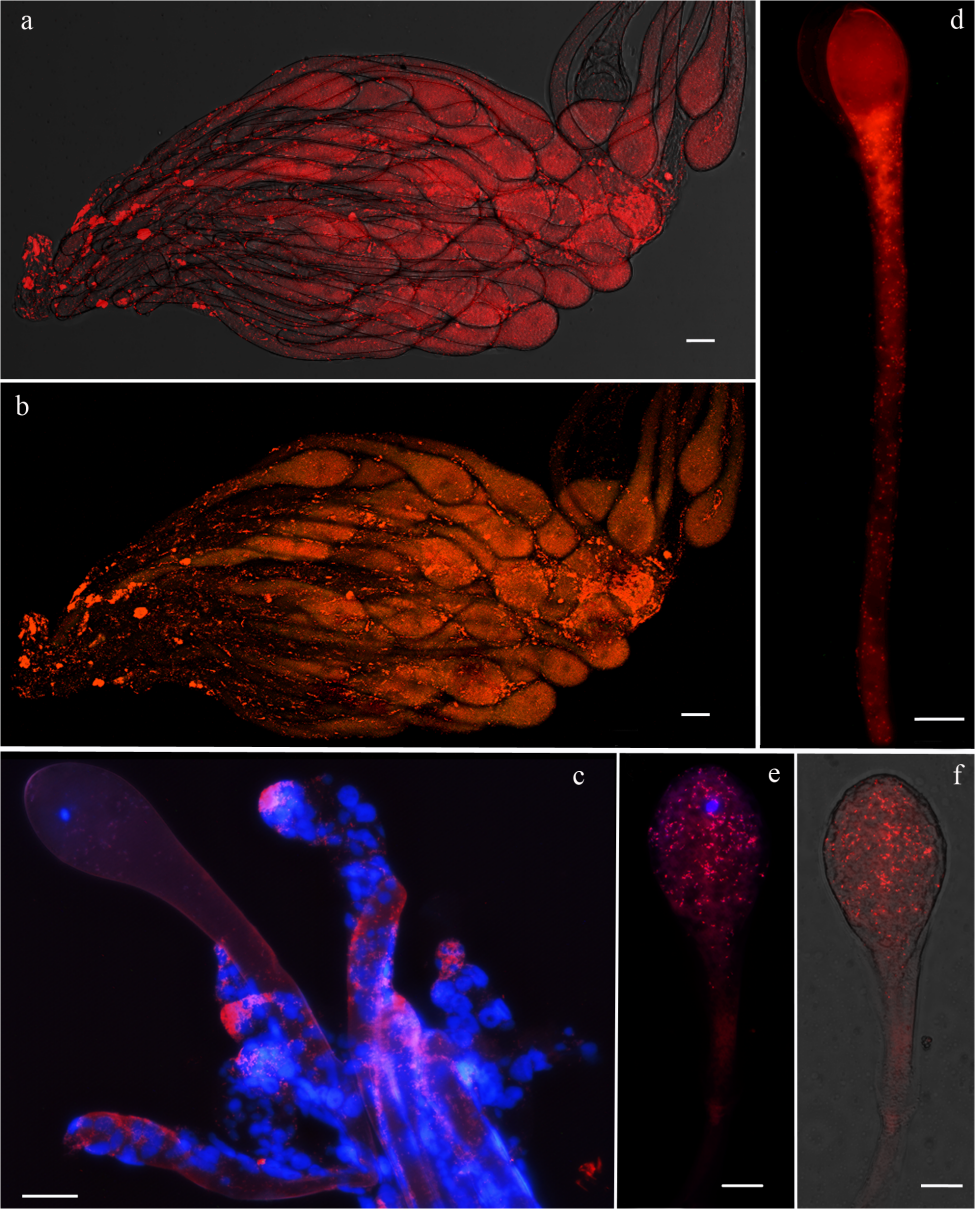
FISH on *L*. *invasa* ovarioles and eggs. Distribution of *Rickettsia* in the ovary (A and B), germaria (C) and the eggs (D, E, and F) of *Leptocybe invasa*. In the ovary *Rickettsia* bacteria (bright red spots) are inside the developing oocytes (A) and are densely clustered in the germaria (C). Merged image of ovarioles simultaneously stained with the *Rickettsia* specific probe RickPn-Cy3 and the universal bacterial probe EUB338-Cy5, showing *Rickettsia* cells in orange (B). Within the egg, bacteria are distributed in the head (E and F) and in the peduncle (D). DAPI-stained nuclei are blue. Bars, 20 μm.

Using FISH analysis, we found that *Rickettsia* occur in the reproductive tissues of *L*. *invasa* females. Inside the ovary, dense clusters of bacteria were observed in the ovarioles (Fig 3A) and in the germarium (Fig 3C). Simultaneous probing with the *Rickettsia* specific probe and the universal bacterial probe (S4A and S4B Fig) did not reveal the occurrence of different bacteria out of *Rickettsia* (Fig 3B) showing a full overlay of two probes. Within the developed oocytes, bacteria were densely distributed both near the nucleus (Fig 3D and 3E) and in the peduncle (Fig 3F). Negative controls (no-probe and RNase-digested controls) did not display any signal, confirming the specificity of the detected signals.

## Discussion

Specimens of *L*. *invasa* from different countries here examined all correspond to a single morphospecies. However, the integration of phylogenetic analysis, bPTP species delimitation method, statistical parsimony, COI distances and diagnostic SNPs, genetic differences at the strain level of *Rickettsia* symbionts, and sex ratio differences supports separation of the *Chinese* and *Western* lineages, which can be therefore treated as putative species.

Indeed, two genetically distinct lineages of *L*. *invasa* were identified on the basis of molecular, phylogenetic and species delimitation analyses. One lineage included populations from the Mediterranean region and South America, the other was present in China. The two lineages showed differences in all sequenced genes. Whereas ITS2 resulted to be more conserved than in other chalcidoid taxa, variation in COI and 28S-D2 is compatible with species level divergence reported previously. Indeed, 28S-D2 is often invariant between closely related species that are instead well differentiated on the base of other genes (usually COI) and biological traits [70,45]. In one case, even Palearctic and Nearctic species of the genus *Pnigalio* (Eulophidae) shared the same 28S-D2 sequence [45]. The single 28S-D2 polymorphism in *L*. *invasa* is consistent with what already observed in Eulophidae, where a high variation in COI can correspond to just a single diagnostic polymorphism in 28S-D2 [36]. As for COI, the divergence (<4%) is similar to that between two species pairs of the genus *Encarsia* (Aphelinidae) (Gebiola et al. in prep). The lack of COI genetic variation in specimens from the same locality (with the exception of the Turkish population) could be explained either by founder effects (the reduced genetic variation that occurs when a population is established starting from a few or a single specimen) [71], or by endosymbiont infection. Endosymbionts, acting as reproductive manipulators, are considered responsible for the very low mitochondrial genetic diversity in infected populations [32, 72], indeed they can induce selective sweeps that indirectly impact the existing polymorphism of mtDNA [73,74].

Among the known reproductive manipulators, *Rickettsia* was the only one found to infect *L*. *invasa*. Phylogenetic reconstructions based on three different genes (16S rRNA, *gltA* and *atpA*) placed this symbiont within the *Rickettsia* transitional group, in a clade that includes the symbionts of the eulophid wasps *Pediobius rotundatus* (Fonscolombe) and *N*. *formosa* [68]. Congruently, the *rpmE*-tRNAf^Met^ gene sequence showed the highest similarity with *R*. *felis*, a species also belonging to the transitional group [68,75]. In particular, *Rickettsia* gene sequence analysis revealed the occurrence of two closely related bacterial strains, one associated with the putative *Western* species and one with the putative *Chinese* species. The 16S rDNA, *gltA*, *atpA* and *rpmE*-tRNAf^Met^ sequences of the symbionts of the *Western* species samples showed no differences with the exception of 16S rRNA gene sequence of the Turkish specimen Li-TK2, differing by two nt. Instead, the *Rickettsia* strain associated with the *Chinese* species differed from the *Western* species in three out of four genes (16S rRNA, *gltA* and *rpmE*-tRNAf^Met^).

The strict association between the two *Rickettsia* strains and the two *L*. *invasa* lineages is also evident by the striking congruence between symbiont and host phylogenetic trees (S3 Fig), which suggests strict vertical transmission of the symbiont. Congruent topologies of host and symbiont molecular phylogenies are consistent with coevolution, but could be caused by other factors as well, such as resource tracking, where the symbiont evolves in response to a host trait with a phylogenetic signal [76].

Populations of *L*. *invasa* are known to have different sex ratios over their geographic distribution. Males have never been recorded from Italy ([1], see also S2 Table), Tunisia [34] and Argentina [35], whereas males are rare in Turkey (sex ratio 0–0.5%) [21]. In these countries, it is clear that *L*. *invasa* reproduces by thelytokous parthenogenesis. In contrast, a high frequency of males has been recorded in populations from China (males ranging from 18–48% in some populations) [17,26] and in populations from South–East Asia (Taiwan, India, Thailand) [22,23,24,25]. Interestingly, the genetic differentiation within *L*. *invasa* shown here mirrors the geographic variation in sex ratio. However, despite the occurrence of males, there is evidence suggesting that some populations from South-East Asia also reproduce by thelytokous parthenogenesis (although biparental populations cannot be excluded). Indeed, populations with only occasional males occur in China as well [77]. Furthermore, virgin females of the Chinese population studied in this work (Wang, unpublished observations) and of a Thai population [24] are able to produce both females and males, with male progeny apparently being not functional [24].

Why do such differences in the sex ratio exist between thelytokous populations of the *Western* and Chinese putative species? Results discussed above and the evidence that *Rickettsia* symbionts of *L*. *invasa* occur at high density within the ovaries and the eggs and is transmitted vertically to the progeny with very high efficiency support the hypothesis that *Rickettsia* can induces thelytokous parthenogenesis in *L*. *invasa*. Removing *Rickettsia* symbionts by feeding females antibiotics should restore the production of male progeny [33,78], but, despite numerous attempts, we were not able to rear and thus cure infected *L*. *invasa*. Furthermore, the interaction between host and symbiont genotype plays an important role in the phenotypic effect and transmission efficiency of reproductive manipulators [79]. Low transmission efficiency of *Rickettsia* could make the sex ratio of thelytokous individuals more susceptible to the effect of environmental factors. Efficiency of symbiont transmission through the host germline, but also penetrance of the reproductive phenotype, and infection prevalence in the host population, may be correlated with bacterial density [80]. Bacterial density is in turn regulated by genetic factors of the host and the symbiont itself and is strongly influenced by environmental factors, like temperature, antibiotics, and host age [81,82]. The general variation in bacterial density in response to temperatures indicates that there can be large spatial, temporal, and seasonal differences in endosymbiont densities and functions in natural populations. High temperatures, by reducing the symbiont density within the reproductive tissues of the host, can induce the production of male progeny by infected parthenogenetic females and variable sex ratios in field populations [83,84]. In the case of *L*. *invasa*, laboratory experiments with a Chinese population have shown that at constant temperatures the frequency of males in the progeny of thelytokous females increases from 2% at 20–23°C to 7% around 30°C [77]. Therefore, it is possible that the association between *Rickettsia* strain and its Chinese host is weaker (in terms of bacterial density and/or transmission efficiency) than that occurring with its *Western* host, and consequently more susceptible to the effect of high and/or low temperatures. Another plausible explanation for the different sex ratios of the Chinese species may be a more recent origin of the symbiotic association with *Rickettsia* than in the *Western* species. Thus, the production of frequent male offspring by infected Chinese females may be due to a maladaptive side effect of incomplete coevolution between host and symbiont, as recorded in another host—parasitoid system [85]. Besides, a possible influence of the host plant cannot be excluded, as the Chinese specimens were collected on *E*. *globulus* instead of *E*. *camaldulensis*. It has been demonstrated in other systems that plants on which phytophagous insects feed may influence the host-symbiont relationship [86,87]. Lastly, unless a direct involvement of *Rickettsia* in the thelytokous reproduction [33,78] or in the oogenesis [88] of *L*. *invasa* is conclusively demonstrated (by obtaining males or no eggs respectively from cured females), we cannot rule out the possibility that thelytoky could be genetically determined.

The likely existence of more than one species behind the morphospecies *L*. *invasa* could have important implications in terms of pest management. For example, parasitoids introduced in several countries [19] could have a different degree of specificity towards the two cryptic species, being able to parasitize only one of them or performing sub-optimally on different host species. It is therefore very important to determine whether the host range of the main parasitoid species described as monophagous (*Selitrichodes kryceri* Kim & La Salle, *Selitrichodes neseri* Kelly & La Salle, and *Quadrastichus mendeli* Kim & La Salle) [4,89] includes both cryptic species of *L*. *invasa*. Moreover, an influence on parasitoid performance might be caused not just by different hosts but also by the presence of diverse bacteria, as recently showed in other systems where different species of endosymbionts, or even slightly different strains, have an impact on the specificity of host—parasitoid interactions [90–93].

## Concluding Remarks

We showed that the world wide distribution of *L*. *invasa* very likely involves at least two species showing distinct sex-ratios, that *Rickettsia* may be the causal agent of thelytokous reproduction, that two different symbiont strains are associated with the two putative host species, and that the host evolutionary history is recapitulated in the relationship of their microbial symbionts. Based on these results, a better understanding of the interaction between the host and symbiont is critical to explain biological differences. Furthermore, a deeper knowledge and characterization of the different populations of *L*. *invasa* from around the world is an essential challenge that should be addressed because of its consequences on pest management. As *L*. *invasa* is now widespread and biological control seems to be the best solution of its management, it is important to reassess the efficiency of the parasitoids currently used on both cryptic gall wasps, in order to avoid failures of biological control programs.

## Supporting Information

S1 Fig Bayesian majority-rule consensus tree based on a concatenated dataset of ITS2, 28S-D2 and COI sequences of *L*. *invasa*. The evolutionary model selected by MrModeltest2 was GTR+G. Scale bar indicates the number of substitutions per site.
(TIF)

S2 Fig Phylogenetic placement of the symbiont from *L*. *invasa* within the genus *Rickettsia*. (a) Bayesian phylogeny based on *atpA* sequences (Evol. model: GTR+I+G); (b) Bayesian phylogeny based on *gltA* sequences (Evol. model: TVM+G). The host is provided whenever the symbiont is not identified at the species level. Posterior probabilities are reported above branches. Scale bar indicates the number of substitutions per site.
(TIF)

S3 Fig Tanglegram showing congruence between host and symbiont ML trees. Bootstrap values (>75%) are reported above branches. Scale bar indicates the number of substitutions per site.
(TIF)

S4 Fig FISH on *L*. *invasa* ovarioles. *Rickettsia* bacteria, stained with *Rickettsia* specific probe RickPn-Cy3, appear like bright red spots on the ooplasm background (A), while appear like bright green spots on the ooplasm background (B) when stained with universal bacterial probe EUB388-Cy5. Bars, 20 µm.
(TIF)

S1 File Uncorrected (a) intra- and (b) inter-lineage p-distances calculated on the COI dataset. (DOCX)

S1 Table First record of *Leptocybe invasa* in invaded Countries, with bibliographic references. *Presence of males.
(DOCX)

S2 Table Sex and collection date of *L*. *invasa* specimens sampled in Italy. (DOCX)

S3 Table Diagnostic nucleotide positions in the COI alignment for the *L*. *invasa* specimens involved in the analyses. (DOCX)
