# Supplementary figures and images for: Genetic Diversity of the Invasive Gall Wasp Leptocybe invasa (Hymenoptera: Eulophidae) and of its Rickettsia Endosymbiont, and Associated Sex-Ratio Differences
Source: PLoS One. 2015 May 13;10(5):e0124660. doi: 10.1371/journal.pone.0124660 (PMC4430503; doi:10.1371/journal.pone.0124660)

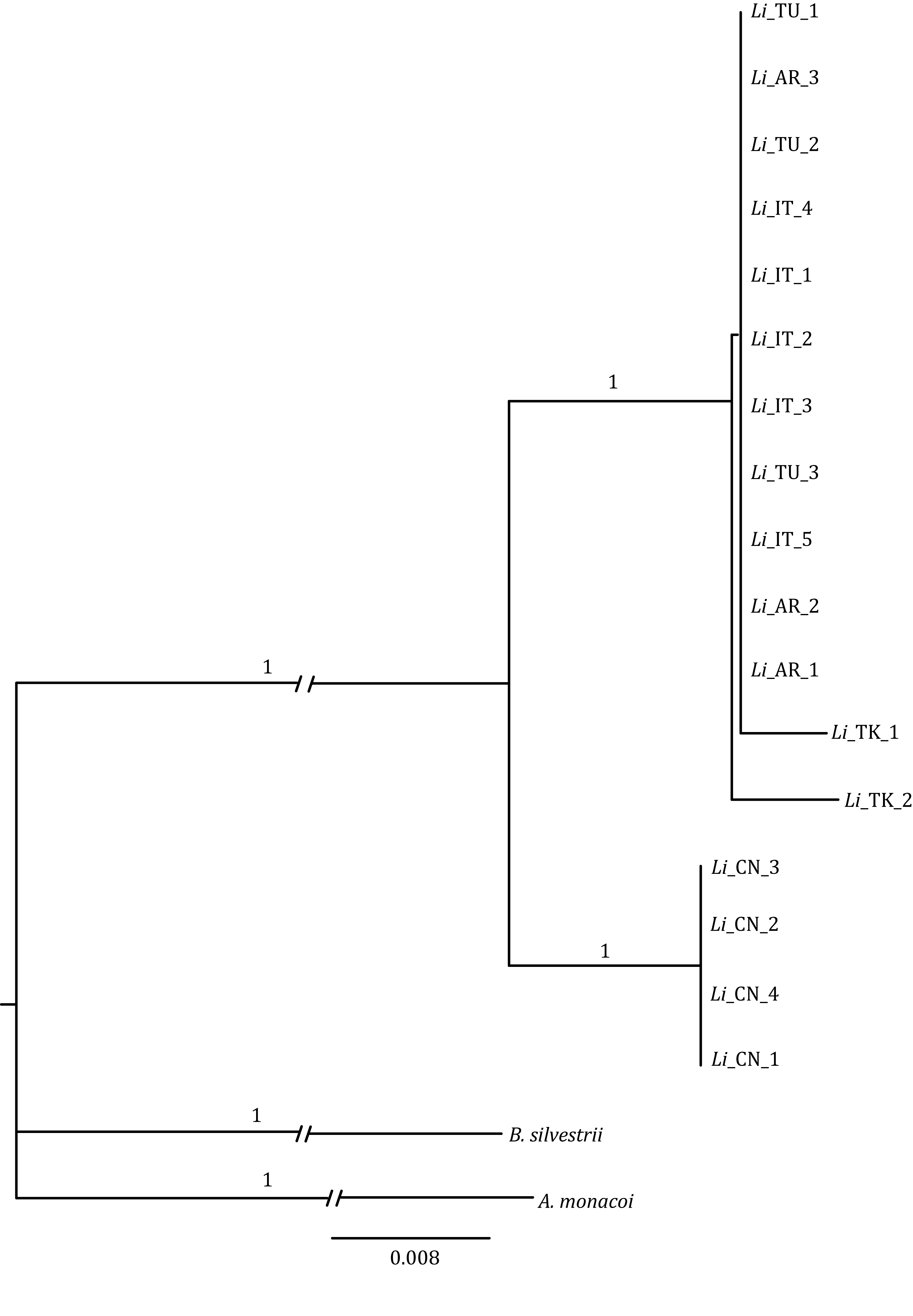

Supplement: S1 Fig — The evolutionary model selected by MrModeltest2 was GTR+G. Scale bar indicates the number of substitutions per site. (TIF) [file pone.0124660.s001.tif]

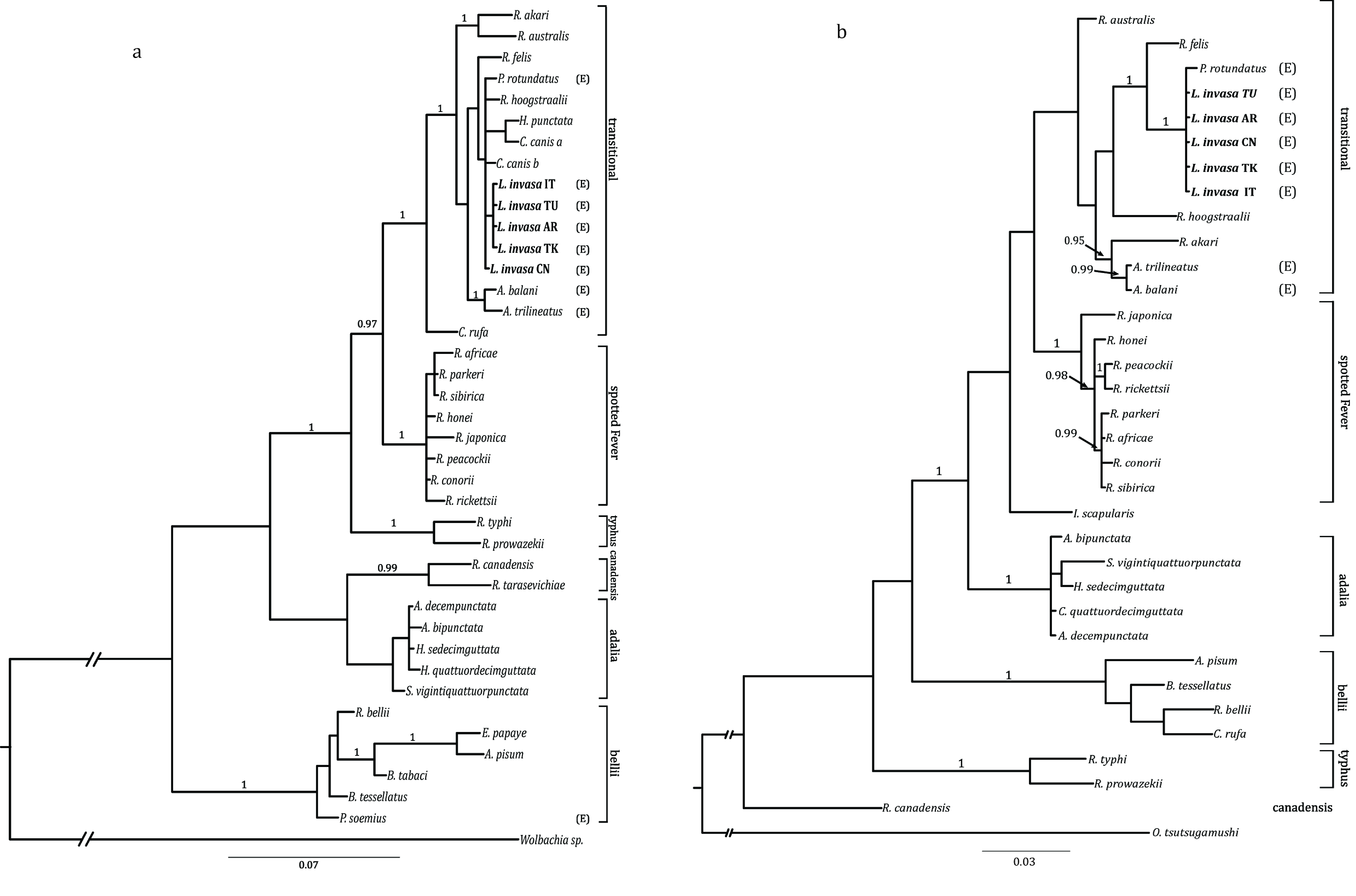

Supplement: S2 Fig — (a) Bayesian phylogeny based on atpA sequences (Evol. model: GTR+I+G); (b) Bayesian phylogeny based on gltA sequences (Evol. model: TVM+G). The host is provided whenever the symbiont is not identified at the species level. Posterior probabilities are reported above branches. Scale bar indicates the number of substitutions per site. (TIF) [file pone.0124660.s002.tif]

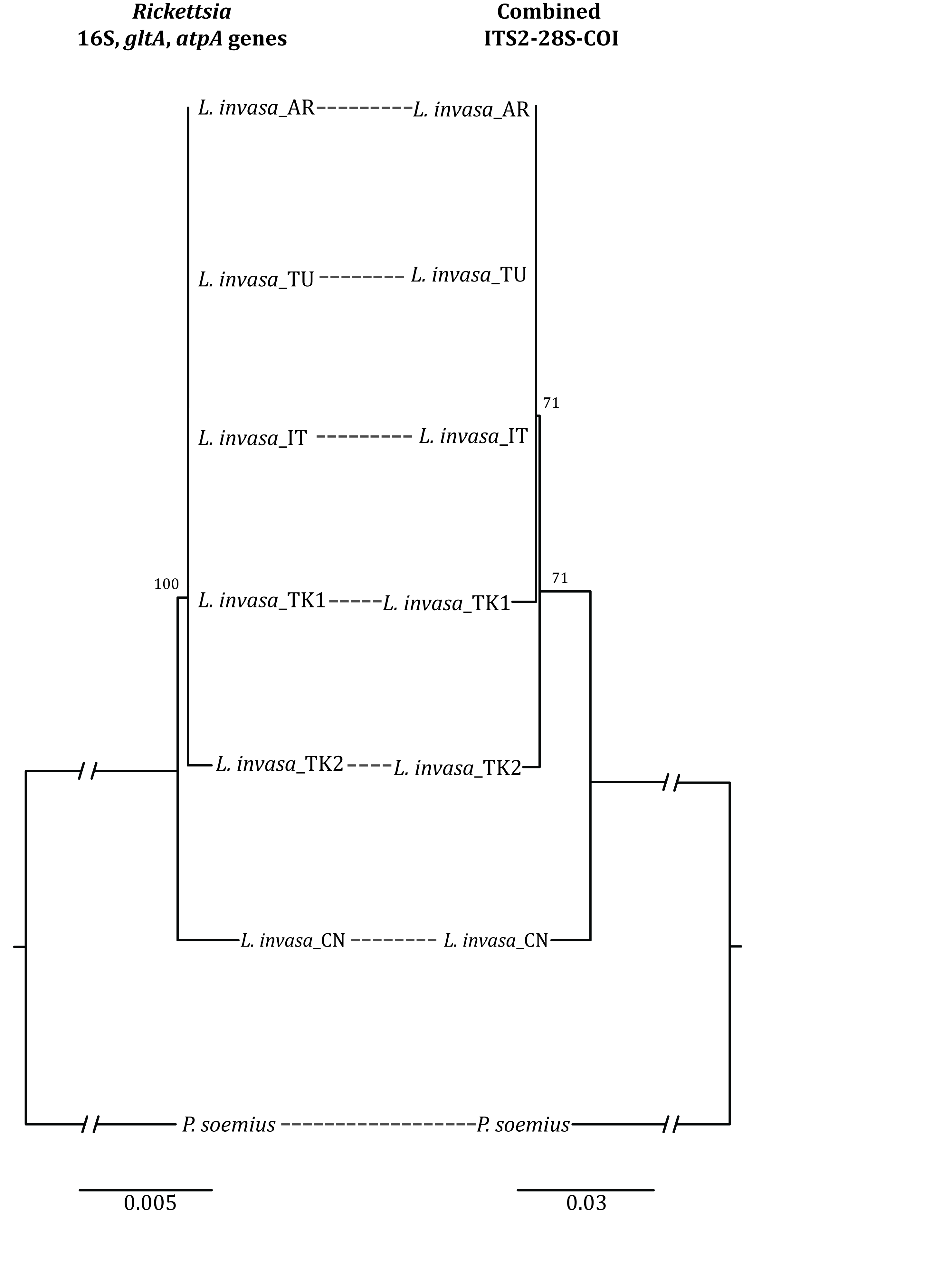

Supplement: S3 Fig — Bootstrap values (>75%) are reported above branches. Scale bar indicates the number of substitutions per site. (TIF) [file pone.0124660.s003.tif]

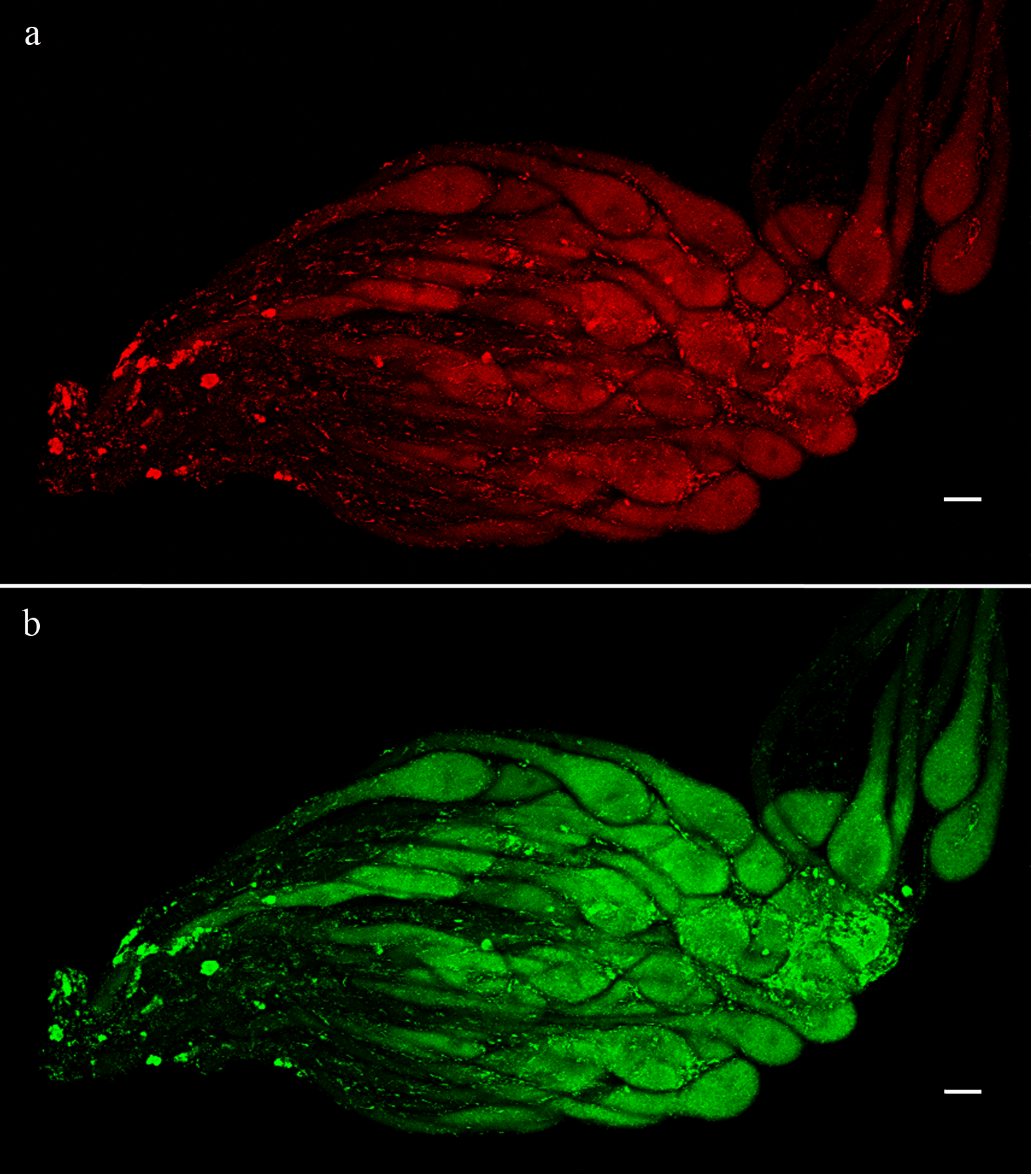

Supplement: S4 Fig — Rickettsia bacteria, stained with Rickettsia specific probe RickPn-Cy3, appear like bright red spots on the ooplasm background (A), while appear like bright green spots on the ooplasm background (B) when stained with universal bacterial probe EUB388-Cy5. Bars, 20 µm. (TIF) [file pone.0124660.s004.tif]
