# Supplementary material for: Genetic Diversity of the Invasive Gall Wasp Leptocybe invasa (Hymenoptera: Eulophidae) and of its Rickettsia Endosymbiont, and Associated Sex-Ratio Differences
Source: PLoS One. 2015 May 13;10(5):e0124660. doi: 10.1371/journal.pone.0124660 (PMC4430503; doi:10.1371/journal.pone.0124660)
Supplement: S1 File — (DOCX) [file pone.0124660.s005.docx]

**S1 File. Uncorrected (Tables 1-2) inter- and (Table 3) intra-lineage p-distances and standard errors (in italic) calculated on the COI dataset.**

Table 1

|  | *A. monacoi* | *L. invasa* lineage A | *L. invasa* CN | *L. invasa* TK | *B. silvestrii* |
| --- | --- | --- | --- | --- | --- |
| *A. monacoi* |  | *0.009* | *0.009* | *0.009* | *0.009* |
| *L. invasa* lineage A | 0.154 |  | *0.005* | *0.001* | *0.009* |
| *L. invasa* CN | 0.150 | 0.031 |  | *0.005* | *0.009* |
| *L. invasa* TK | 0.156 | 0.007 | 0.037 |  | *0.009* |
| *B. silvestrii* | 0.137 | 0.130 | 0.133 | 0.135 |  |

Table 2

|  | *L. invasa* Western lineage | *L. invasa* Chinese lineage |
| --- | --- | --- |
| *L. invasa* Western lineage |  | *0.005* |
| *L. invasa* Chinese_lineage | 0.036 |  |

Table 3

|  | d |  |
| --- | --- | --- |
| *A. monacoi* | n/c | *n/c* |
| *L. invasa* lineage A | 0.0000 | *0.0000* |
| *L. invasa* CN | 0.0000 | *0.0000* |
| *L. invasa* TK | 0.0147 | *0.0030* |
| *B. silvestrii* | n/c | *n/c* |
