# Supplementary material for: Genetic Diversity of the Invasive Gall Wasp Leptocybe invasa (Hymenoptera: Eulophidae) and of its Rickettsia Endosymbiont, and Associated Sex-Ratio Differences
Source: PLoS One. 2015 May 13;10(5):e0124660. doi: 10.1371/journal.pone.0124660 (PMC4430503; doi:10.1371/journal.pone.0124660)
Supplement: S1 Table — *Presence of males. (DOCX) [file pone.0124660.s006.docx]

**S1 Table. First record of *Leptocybe invasa* in invaded Countries, with bibliographic references.**

| **First record** | **Country** | **Reference** |
| --- | --- | --- |
| 2000 | Italy (including Sicily) | [1] |
|  | Turkey* | [2] |
| 2001 | India (Karnataka)* | [94] |
|  | Israel, Morocco | [3] |
|  | Egypt, Jordan, Syria | [16] |
| 2002 | Algeria | [3] |
|  | Kenya | [95] |
|  | Uganda | [96] |
|  | Ethiopia | [97] |
|  | Vietnam | [98] |
| 2003 | Spain (including Balearic Islands) | [99] |
|  | Portugal | [100] |
| 2004 | Corsica (France), Greece | [16] |
|  | Tunisia | [34] |
|  | Thailand* | [19] |
| 2005 | France | [101] |
|  | Iran | [102] |
|  | Tanzania | [103] |
| 2006 | Southern England | [16] |
|  | Malta | [104] |
| 2007 | Zimbabwe | [105] |
|  | South Africa | [106] |
|  | China* | [107] |
|  | Brazil | [108] |
| 2008 | United State of America (Florida) | [109] |
|  | Malawi | [110] |
|  | Laos | [20] |
|  | Australia (indirect) | [4] |
| 2009 | Argentina | [35] |
| 2010 | Chile | [16] |
|  | Iraq | [111] |
|  | Sri Lanka | [112] |
|  | Taiwan | [25] |
| 2012 | Malaysia | [19] |
|  | Mozambique | [113] |

*Presence of males

1. Kavitha UN. Bioecology and management of eucalyptus gall wasp*, Leptocybe invasa* Fisher & La salle (Hymenoptera: Eulophidae). Master's Thesis, University of Agricultural Sciences, Bangalore, India. 2010
2. Mutitu KE. A pest threat to eucalyptus species in Kenya. KEFRI Technical Report. 2003;p. 12.
3. Nyeko P, Mutitu KE, Day RK. Eucalyptus infestation by *Leptocybe invasa* in Uganda Afr. J. Ecol., 2009;47: 299–307
4. Giliomee JH. Recent establishment of many alien insects in South Africa, a cause for concern. African Entomology. 2011;19: 151-155.
5. Thu PQ. The first record of gall forming wasp associated with eucalypt plantations in Vietnam. Sci Tech J Agr Rural Dev **11**, 1598–9.
6. Sánchez I (2003) Descubiertas dos nuevas plagas del eucalipto en España. Quercus, 214: 32-33.
7. Branco M, Franco JC, Valente C, Mendel Z. Survey of Eucalyptus gall wasps (Hymenoptera: Eulophidae) in Portugal. Bol. Sanidad Veg Plagas. 2006;32: 199-202.
8. EPPO. Reporting service n.6. Paris, France: European and Mediterranean Plant Protection Organization. 2006. Available: <https://archives.eppo.int/EPPOReporting/2006/Rse-0609.pdf>
9. Hesami S, Alemansoor H, Seyedebrahimi S. Report of *Leptocybe invasa* (Hym.: Eulophidae), Gall Wasp of *Eucalyptus camaldulensis* with Notes on Biology in Shiraz Vicinity. J Entomol. Soc. IRAN. 2005;24: 99-108.
10. Roux J (Ed.). Pest alert. Blue gum chalcid. *Tree Protection News*. 2005;10: 13. Available: www.fabinet**.**up**.**ac**.** za**/**tpcp**/**Leptocybe_alert).
11. Mifsud D. *Leptocybe invasa* Fisher & La Salle, 2004 and *Ophelimus maskelli* Haliday, 1844-two new records of gall forming Eulophidae from Malta (Hymenoptera, Chalcidoidea). Bull. Ent. Soc. Malta. 2012;5: 189–193.
12. Ministry of environment & natural resources management. Zimbabwe’s fourth national report to the convention on biological diversity. MENRM, Harare, Zimbabwe. 2010. Available: [www.cbd.int/doc/world/zw/zw-nr-04-en.pdf](http://www.cbd.int/doc/world/zw/zw-nr-04-en.pdf)
13. Dittrich-Schröder G, Wingfield MJ, Hurley B, Neser, S, Mendel Z, Slippers B. The invasive gall-forming wasp *Leptocybe invasa* (Hymenoptera: Eulophidae) in South Africa. *Proceedings of the 16th* Congress of the Entomological Society of Southern Africa, Stellenbosch. Entomol. Soc. S. Africa, Stellenbosch 2009;27
14. Wu YJ, Jiang XJ, Li DW, Luo JT, Zhou GF, Chang MS, et al. *Leptocybe invasa*, a new invasive forest pest making galls on twigs and leaves of eucalyptus trees in China. *Sci. Silvae Sin*. 2009;45: 161-163.
15. Costa VA, Berti Filho E, Wilcken CF, Stape JL. Eucalyptus gall wasp, *Leptocybe invasa* Fisher & La Salle (Hymenoptera: Eulophidae) in Brazil: New forest pest reaches the New World. Revista de Agricultura (Piracicaba) 2008;83: 136-139.
16. Wiley J, Skelley P. A *Eucalyptus* pest, *Leptocybe invasa* Fisher & La Salle (Hymenoptera: Eulophidae), genus and species new to Florida and North America. Pest Alert. Florida Department of Agriculture & Consumer Services. 2008. Available: <http://www.freshfromflorida.com/Divisions-Offices/Plant-Industry/Plant-Industry-Publications/Pest-Alerts/Pest-Alerts-A-Eucalyptus-Pest-Leptocybe-Invasa>
17. FRIM (Forest Research Institute of Malawi) Biodiversity in Malawi. 2010. Available: http://www.chmmw.org/ biodivmw.asp
18. Hassan FR. First record of the eucalyptus gall wasp, *Leptocybe invasa* Fisher and la Salle (Hymenoptera: Eulophidae), in Iraq. Acta Agrobotanica. 2012;65: 1-6.
19. Karunaratne WAIP, Edirisinghe JP, Ranawana KB. Rapid survey of damage due to gall wasp infestation in a coppiced *Eucalyptus camaldulensis* plantation in Maragamuwa, Naula in the Matale District of Sri Lanka. *Ceylon J. Sc. (Biological Sciences)* 2010;39: 157-161
20. IPPC (International Plant Protection Convention). Occurrence of eucalyptus gall wasp *Leptocybe invasa* in Mozambique. 2012. Available: <https://www.ippc.int/fr/content/occurence-eucalyptus-gall-wasp-leptocybe-invasa-mozambique>
