# Supplementary material for: Genetic Diversity of the Invasive Gall Wasp Leptocybe invasa (Hymenoptera: Eulophidae) and of its Rickettsia Endosymbiont, and Associated Sex-Ratio Differences
Source: PLoS One. 2015 May 13;10(5):e0124660. doi: 10.1371/journal.pone.0124660 (PMC4430503; doi:10.1371/journal.pone.0124660)
Supplement: S2 Table — (DOCX) [file pone.0124660.s007.docx]

| **Date of collection** | **Specimens collected** | **Females** | **Males** | **Locality** |
| --- | --- | --- | --- | --- |
| 31.05.13 | 3 | 3 | 0 | Portici (NA) |
| 01.06.13 | 13 | 13 | 0 | Portici (NA) |
| 02.06.13 | 29 | 29 | 0 | Portici (NA) |
| 03.06.13 | 18 | 18 | 0 | Portici (NA) |
| 04.06.13 | 13 | 13 | 0 | Portici (NA) |
| 05.06.13 | 21 | 21 | 0 | Portici (NA) |
| 06.06.13 | 7 | 7 | 0 | Portici (NA) |
| 07.06.13 | 37 | 37 | 0 | Portici (NA) |
| 08.06.13 | 43 | 43 | 0 | Portici (NA) |
| 09.06.13 | 41 | 41 | 0 | Portici (NA) |
| 10.06.13 | 141 | 141 | 0 | Portici (NA) |
| 11.06.13 | 38 | 38 | 0 | Portici (NA) |
| 12.06.13 | 20 | 20 | 0 | Portici (NA) |
| 13.06.13 | 15 | 15 | 0 | Portici (NA) |
| 15.06.13 | 57 | 57 | 0 | Portici (NA) |
| 18.06.13 | 11 | 11 | 0 | Portici (NA) |
| 19.06.13 | 13 | 13 | 0 | Portici (NA) |
| 20.06.13 | 21 | 21 | 0 | Palma Campania (NA) |
| 21.06.13 | 15 | 15 | 0 | Portici (NA) |
| 22.06.13 | 22 | 22 | 0 | Portici (NA) |
| 27.06.13 | 2 | 2 | 0 | Portici (NA) |
| 01.18.13 | 22 | 22 | 0 | Costa Saracena (SR) |
| 16.10.13 | 24 | 24 | 0 | Portici (NA) |
| 17.10.13 | 25 | 25 | 0 | Portici (NA) |
| 18.10.13 | 4 | 4 | 0 | Portici (NA) |
| 25.10.13 | 4 | 4 | 0 | Portici (NA) |
| 28.10.13 | 4 | 4 | 0 | Portici (NA) |
| 29.10.13 | 1 | 1 | 0 | Portici (NA) |
| 05.11.13 | 58 | 58 | 0 | Portici (NA) |
| 10.6.14 | 6 | 6 | 0 | Orosei (NU) |
| 07.07.14 | 260 | 260 | 0 | Portici (NA) |
| 22.7.14 | 19 | 19 | 0 | Santa Maria al Bagno (LE) |
| 14.08.14 | 34 | 34 | 0 | Portici (NA) |
| 08.09.14 | 46 | 46 | 0 | Portici (NA) |
| 17.09.14 | 1 | 1 | 0 | Portici (NA) |
| 19.09.14 | 2 | 2 | 0 | Portici (NA) |
| 23.09.14 | 5 | 5 | 0 | Portici (NA) |
| 24.09.14 | 4 | 4 | 0 | Portici (NA) |
| 27.09.14 | 3 | 3 | 0 | Portici (NA) |
| 28.09.14 | 1 | 1 | 0 | San Giorgio a Cremano (NA) |
| 01.10.14 | 1 | 1 | 0 | San Giorgio a Cremano (NA) |
| 02.10.14 | 1 | 1 | 0 | San Giorgio a Cremano (NA) |
| 01.10.14 | 1 | 1 | 0 | San Giorgio a Cremano (NA) |
| 02.10.14 | 1 | 1 | 0 | San Giorgio a Cremano (NA) |
| 10.10.14 | 3 | 3 | 0 | San Giorgio a Cremano (NA) |
| 15.10.14 | 2 | 2 | 0 | Portici (NA) |
| 20.10.14 | 5 | 5 | 0 | San Giorgio a Cremano (NA) |
| 17.11.14 | 12 | 12 | 0 | Roma |
| **Total** | **1129** | **1129** | **0** |  |
|  | **Specimens** | **Females** | **Males** |  |

**S2 Table. Sex and collection date of *L. invasa* specimens sampled in Italy.**
