# Supplementary material for: Genetic Diversity of the Invasive Gall Wasp Leptocybe invasa (Hymenoptera: Eulophidae) and of its Rickettsia Endosymbiont, and Associated Sex-Ratio Differences
Source: PLoS One. 2015 May 13;10(5):e0124660. doi: 10.1371/journal.pone.0124660 (PMC4430503; doi:10.1371/journal.pone.0124660)
Supplement: S3 Table — (DOCX) [file pone.0124660.s008.docx]

| COI Diagnostic nucleotide position | | | | | | | | | | | | | | | | | | | | | | | | | | | | | | | | | | | | | | | | | | | |
| --- | --- | --- | --- | --- | --- | --- | --- | --- | --- | --- | --- | --- | --- | --- | --- | --- | --- | --- | --- | --- | --- | --- | --- | --- | --- | --- | --- | --- | --- | --- | --- | --- | --- | --- | --- | --- | --- | --- | --- | --- | --- | --- | --- |
| Genetic groups | 040 | 066 | 078 | 084 | 093 | 147 | 201 | 228 | 231 | 232 | 234 | 237 | 240 | 285 | 309 | 342 | 351 | 453 | 525 | 609 | 642 | 717 | 780 | 852 | 888 | 942 | 952 | 969 | 985 | 1005 | 1026 | 1041 | 1093 | 1098 | 1209 | 1212 | 1224 | 1233 | 1272 | 1372 | 1404 | 1413 | 1451 |
| *L. invasa* Lineage A + Turkey | C | A | T | T | T | A | T | A | G | C | G | C | A | G | T | C | C | A | T | A | C | T | T | A | T | A | C | T | T | C | C | C | A | C | A | G | G | C | T | A | T | C | T |
| *L. invasa* China | T | G | C | C | C | G | C | G | A | T | A | T | T | A | C | T | T | G | A | G | T | C | A | T | C | T | T | A | C | T | T | T | G | T | G | A | A | T | A | T | A | T | C |

S3 Table. Diagnostic nucleotide positions in the COI alignment for the *L. invasa* specimens involved in the analyses.
